# Origin of the trochophora larva

**DOI:** 10.1098/rsos.180042

**Published:** 2018-07-25

**Authors:** Claus Nielsen

**Affiliations:** Biosystematics, The Natural History Museum of Denmark, University of Copenhagen, Universitetsparken 15, Copenhagen, Denmark

**Keywords:** larvae, embryology, development, ciliary bands, blastomere labelling, cell-lineage

## Abstract

The trochophora larva, which is so well known from the marine plankton, is central to our understanding of the evolution of a large branch of the bilaterians. Two theories for this larval type have been prevalent, the trochaea theory and the theory proposed by Ivanova-Kazas. The embryology, or more precisely the cell-lineage, of these larvae seems to be central for our understanding of their origin, but important details have been missing. According to the trochaea theory, a circumblastoporal ring of blastomeres differentiates to follow the convoluted shape of the conspicuous ciliary bands of the larvae, with prototroch and metatroch around the mouth, forming a filtering system, and telotroch around the anus. According to the Ivanova-Kazas theory, the blastomeres with the ciliary bands develop through specialization of rings of cells of the general ciliation in a lecithotrophic larva. Now, a new cell-lineage study of the gastropod *Crepidula* has shown that the ring of cells at the edge of the blastopore develops into the band of cells carrying prototroch and metatroch, characteristic of the trochophora. This gives strong support to the trochaea theory.

## 1. Introduction

**Figure 1.**
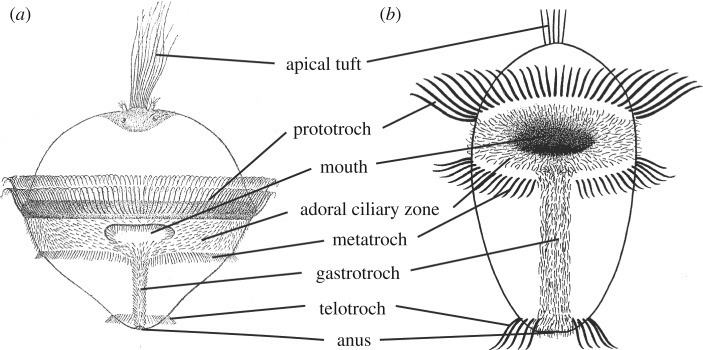
Trochophora concepts. (*a*) Grobben's diagram of a trochophora larva (after [2]). (*b*) A modern diagram of the ciliary bands of a trochophora larva (based on [3]).

Ever since Hatschek [1] introduced the trochophora concept, this larval type has played a prominent role in phylogenetic discussions. In his famous ‘Lehrbuch’ [2], he presented this larval type (figure 1*a*) as the ‘characteristic larval form of the Zygoneura’, i.e. Protostomia, and his diagram is almost identical to a modern concept of the trochophora (figure 1*b*). However, the concept has subsequently been restricted to Lophotrochozoa (*s.l.*) and further to Spiralia *sensu stricto*, i.e. species with spiral cleavage. The typical trochophora larva (figure 1), found in many annelids, molluscs and entoprocts, and the larvae of some platyhelminths (Müller's and Götte's larvae) and nemerteans (pilidium larvae), share important characters with the typical trochophores (reviews in [4,5]). The most characteristic feature of the typical trochophora larva is the ciliary bands, which comprise prototroch, metatroch and telotroch, formed by compound cilia. Prototroch and metatroch are present in all filter-feeding types, but the metatroch is absent in the non-feeding types. Prototroch and metatroch, together with the adoral ciliary zone of separate cilia, function in a downstream-collecting system based on the ‘catch-up principle’ [6]. Ciliary systems of similar structure and function are found in some rotifers and in the cycliophoran *Symbion* [6,7]. The telotroch is locomotory and is found in annelids and aplacophoran molluscs.

The origin of the trochophora larva with its characteristic ciliary bands has been discussed by only a few recent authors. Two main theories are prevalent: the trochaea theory [3,8] and the theory proposed by Ivanova-Kazas [9]. The latter theory is closely related to the ‘planuloid-acoeloid theory’ [10] and the ‘intercalation theory’ [11], but they do not discuss the trochophora larvae.

The morphology of trochophora larvae with its characteristic ciliary bands has been documented in detail in numerous papers, but important details of the embryology have remained controversial, especially the origin of some of the various ciliary bands [5,12–14]. A recent study of the embryology of the snail *Crepidula fornicata,* using blastomere labelling [15], has now filled some of the most important gaps in our knowledge about the embryology, and this new information makes it timely to review the characters and concepts used to infer the origin of this characteristic larval type.

## 2. The trochaea theory

**Figure 2.**
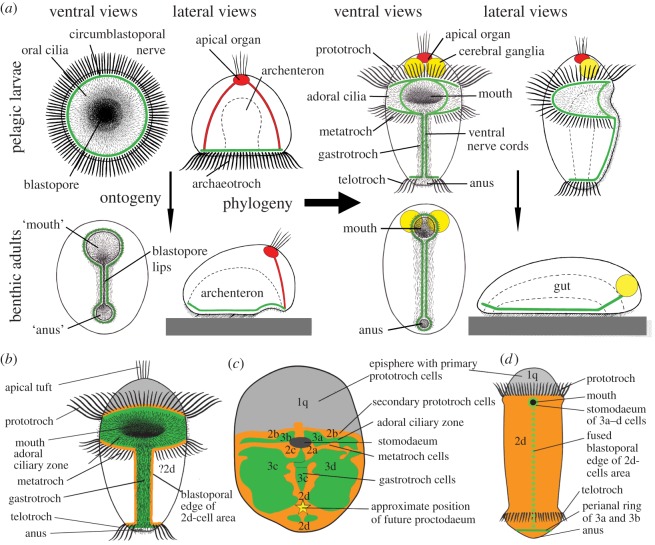
The trochaea theory with its predictions compared with actual cell-lineages. (*a*) The trochaea theory [3]. The two upper left diagrams represent the ancestral trochaea. The four left diagrams together represent a further evolutionary stage, where the larva is planktonic and planktotrophic, with the archaeotroch differentiated into prototroch, metatroch and telotroch, and the adult has become benthic, feeding on deposited material and creating a functional tubular gut by pressing the lateral blastopore lips together. The right diagrams represent the ancestral bilaterian with a trochophora larva and a benthic adult, both with a tubular gut (from [3]). (*b*) The predictions about ciliary bands and cell-lineages of the first, second and third micromere quartets on the surface of the embryo. Cells of the second micromere quartet orange, cells of the third micromere green. (*c*) Cell-lineage of the gastropod *Crepidula*, based on [15]. Cells of the second micromere quartet orange, cells of the third micromere quartet green. (*d*) Cell-lineage of the annelid *Capitella*, based on [16]. This larva is lecithotrophic and accordingly has no metatroch. The study of *Capitella* reported only few gastrotroch cells from the third micromere quartet, but an older study of the annelid *Scoloplos* [17] reported that the whole gastrotroch should originate from third micromere cells (added as the dashed line in the diagram). In *c* and *d*, the positions of prototroch, metatroch and telotroch at the blastoporal edge of the second micromere quartet agree exactly with the trochaea theory, as does the position of third micromere cells around the mouth, along the ventral midline and around the anus.

The trochaea theory (figure 2*a*) [3,8,18] explains the origin of the general morphology and in particular of the ciliated bands (and the associated ventral central nervous system (CNS)) of the trochophora larva through modification of a gastraea-like ancestor called trochaea with a circumblastoporal ring of compound cilia, the archaeotroch (associated with a nervous concentration). The archaeotroch of this holoplanktonic organism, together with a circumblastoporal area of single cilia, should have functioned as a downstream-collecting system using the ‘catch-up’ principle [6]. A similar ring-shaped system is found in the cycliophoran *Symbion* [7]*.* Adults of a trochaea should have taken up a benthic lifestyle, feeding on deposited particles; the band of compound cilia became superfluous and disappeared, but it was retained in the larvae. With the new lifestyle, the adult developed a preferred creeping direction and a new main axis parallel with the substratum. The blastopore lips were pressed together, leaving the anterior mouth and posterior anus, and this gave way to a one-way transport through a tubular digestive tract. This development soon became established already in the larva (through the process called adultation [19]). This type of blastopore closure, called amphistomy, is observed directly in the annelid *Hydroides*, where the posterior opening finally closes, but a new anus develops in the same region [20] and with modifications in many spiralians [4,5]. In the larva, the anterior part of the archaeotroch was retained around the mouth as the prototroch + metatroch (on the anterior and posterior sides of the adoral ciliary zone, respectively), functioning as a downstream-collecting system (see below), and the posterior part remaining around the anus as the telotroch. The circumblastoporal nervous concentration of the trochaea followed the differentiation of the archaeotroch and became the ventral CNS, with paired (or fused) ventral nerve cords with perioral and perianal loops.

Spiralian groups, such as annelids, molluscs, entoprocts, nemerteans and platyhelminths, show a characteristic cleavage pattern known as spiral, and both ‘classical’ and modern cell-lineage studies are available for a number of species with trochophora larvae [14–16] (reviews of the older literature in [4,5]). The cleavage proceeds through a strict pattern which is common to all the phyla. The individual blastomeres in the spiral pattern have been named, and their fates and the origin of several organ systems can be followed from specified cells or groups of cells (figure 2*c*). The ‘classical’ studies of spiralian embryology relied on direct observations of living embryos, and the cleavage patterns, at least of the cells at the surface of the embryos, show practically no variation (reviews in [4,5], see also [14]). The only deviation from the general picture seems to be that the 3c- and 3d-cells contribute to the ‘ciliated band’ in the pilidium larva of the nemertean *Cerebratulus* [21]. Modern studies using blastomere marking generally confirm the old picture, e.g. studies on *Crepidula* [13,15], *Platynereis* [22] and *Capitella* [16]. The few studies on gene expression, e.g. on *Patella* [23] and *Hydroides* [20,24,25], are too scattered to contribute to the discussion.

The first two cleavages divide the zygote into four cells called A–D. The third cleavage divides the embryo into an apical ring of four usually small cells and a blastoporal ring of four usually larger cells. The four apical cells are called the first micromere quartet (called 1a–1d or collectively 1q, their descendants are called 1q^1^ and 1q^2^) is referred to as the episphere (figure 2*c*). It carries an apical sensory organ, usually with an apical tuft of cilia, which develops from descendants of the 1q^11^-cells [26]. This organ seems to be characteristic of ciliated larvae, indicating a common origin of primary ciliated larvae [27]. A pair of cerebral ganglia develops from other cells of the episphere (figure 2*a*), often closely apposed to the apical organ, forming a compound apical organ, but in other species at some distance from it. The cerebral ganglia very often carry a pair of eyes.

However, the most conspicuous feature of the trochophora larvae is the prototroch, an equatorial band of compound cilia [28] used in locomotion and in planktotrophic species in particle collection (see below). Its main component develops from the primary prototroch cells (1q^2^-cells), but it may be accompanied by cilia from accessory prototroch cells (1q^12^-cells) at the apical side, and cilia from secondary prototroch cells (2q-cells) on the blastoporal side [14,29]. It must be emphasized that all these cells are present in the spiral cleavage pattern, whether they develop cilia or not. In *Patella*, some of the ciliated prototroch cells deciliate during embryogenesis [29]. Some lecithotrophic species have a prototroch from primary prototroch cells only, but many species have prototrochs consisting of cilia on two or on all three rings of cells [14]. *Capitella teleta* has rings exclusively of primary prototroch cells, a small ciliary ring from the secondary prototroch cells and rings of both primary and accessory cells [16]. The homology of prototrochs is generally not questioned [14,30].

At the fourth cleavage, the four ‘lower’ blastomeres (called 1A–1D), give off the second micromere quartet (blastomeres 2a–2d). These four cells initially lie anterior, lateral and posterior to the blastopore in species with embolic gastrulation, and in corresponding positions in species with epibolic gastrulation. Their descendants may arrange to form a complete ring, or cells of the third micromere quartet may fill the gaps [31].

The gastropods *Ilyanassa obsoleta* [32,33] and *C. fornicata* (figure 2*c*) [13,15] have epibolic gastrulation, but nevertheless develop planktotrophic larvae with a large velum (they lack a telotroch). The anterior 2b-cells form a narrow row of cells (secondary prototroch cells) just posterior to the primary prototroch cells. The anterior parts of the lateral 2a- and 2c-cells (more precisely 2a^1^ and 2c^1^) form a pair of narrow bands carrying the metatroch, situated parallel to the band of 2b-cells, but separated from it by bands of cells from the third micromere quartet (figure 2*c*) [15] (see below).

There have been some discussions [14] about the origin of the metatroch in the annelid *Polygordius*. The classical study by Woltereck [12], who followed the development of live embryos until a very early, still non-feeding trochophora stage, and reported that two cells of the third micromere quartet, *viz.* 3c^1^ and 3d^1^, divide, each giving rise to an anterior pair of cells, which should become part of the ‘lower lip’ of the mouth, and a posterior pair of cells, 3c^1p^ and 3d^1p^, which should become the precursors of the metatroch cells. This has been given much attention in recent discussions [14]. However, as pointed out earlier [5], this interpretation of the posterior cell is a guess which is not supported by any observations. The two cells show a ciliation continuous with that of the mouth [12, fig. 11] and it appears much more likely that the posterior cells are the precursors of the adoral ciliary zone, which is a lateral extension of the oral ciliation. The more median cells, his 2d^2112^ and 2d^122^, could then be the precursors of the metatroch, which would be in agreement with the well-documented cell-lineage of *Crepidula* mentioned above.

A telotroch surrounding the anus is found in larvae of worm molluscs (caudofoveates and solenogasters) and in many annelids. Its cell-lineage has been studied in several annelids with lecithotrophic larvae. In all species studied, it develops on descendants of the 2d-cell [5,16], and their homology is generally accepted [30].

The ventral CNS, with paired ventral nerve cords and loops around mouth and anus, so well known from many protostomian phyla [34], follows the blastopore lips. It has been shown to develop from cells of the second micromere quartet in *Platynereis* and *Capitella* [16,22].

The cells of the third quartet of *Crepidula* [15] (figure 2*c*) and *Ilyanassa* [32,33] contribute to the stomodaeum, the adoral ciliary zone, the gastrotroch and posterior ventral and dorsal areas.

The ciliary bands of nemerteans and platyhelminths, which also have spiral cleavage, are more difficult to interpret. Their main ciliary band is clearly a prototroch, as shown by cell-lineage studies of both nemerteans [21,35,36] and platythelminths [37]. The nemertean larva called pilidium nielseni has a putative telotroch, but its cell-lineage has not been studied [38].

The trochophora larva, defined by its prototroch developing mainly from the first micromere quartet in the spiral cleavage and in planktotrophic species by the ciliary bands differentiating from cells of the second micromere quartet, is thus a very well-defined larval type which is considered ancestral in the Spiralia. The main ciliary bands of some larvae of nemerteans and platyhelminths may be homologous too.

## 3. Ivanova-Kazas’ theory

**Figure 3.**
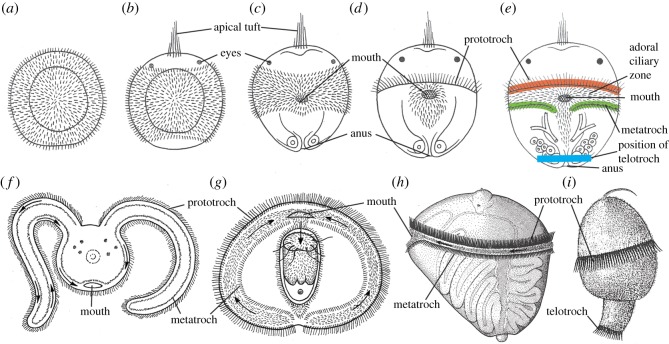
(*a–e*) Stages in the evolution of the trochophore according to Ivanova-Kazas [39]. (*a*) A completely ciliated, lecithotrophic larva. (*b*) A lecithotrophic larva with restricted ciliation, apical tuft and a pair of eyes. (*c*) A larva which has developed mouth and anus (and a gut). (*d*) A ring of (? compound) cilia, a prototroch, has become differentiated in front of the mouth, and most of the general ciliation has been lost. (*e*) A second ring of cilia, a metatroch, has become differentiated behind the mouth, separated from the prototroch by the adoral ciliary zone. The position of the telotroch found in many trochophora larvae has been indicated. The three completely separate ciliary rings, prototroch, metatroch and telotroch, bear no resemblance to the continuous periblastoporal ring of second micromere cells observed in old and new cell lineage studies (figure 2). (*f–i*) Variations of the trochophoran larval type. (*f*) The rostraria larva of an amphinomid annelid, schematic frontal view (after [19]). (*g*) The larva of an undescribed entoproct of the genus *Loxosoma,* ventral view (after [19]). (*h*) The planktotrophic pericalymma larva of the annelid *Polygordius appendiculatus,* lateral view (after [34]). (*i*) The lecithotrophic pericalymma larva of the mollusc *Neomenia carinata*, lateral view (after [34]). The arrows indicate the direction of the captured particles towards the mouth.

This theory [39] is based on the ‘planuloid–acoeloid theory’ [10] and also the ‘intercalation theory’, which poses that the larvae evolved secondarily to facilitate dispersal [11]. Ivanova-Kazas' theory poses that a generally ciliated, non-feeding larva (figure 3*a*) developed a ring-shaped band of cilia around the body with the mouth on the ventral side (figure 3*b,c*). At first, the cilia were short and separate, but later on, the anterior cilia became longer and presumably compound (figure 3*d*). This ciliary band could have improved swimming abilities [40] (figure 3*d,e*). The origin of the mouth is uncertain, but it was mentioned that it could be the blastopore which had moved forwards. The most anterior band should become the prototroch, the general ciliation around the mouth should become the adoral ciliary zone, and a more posterior band should become the metatroch (figure 3*d,e*); the telotroch was not considered.

However, this theory meets several problems which make the theory quite improbable [41]. The direction of the ciliary beat of the metatroch is opposite that of the prototroch and would counteract the swimming; it would have been of negative adaptational value until the whole complex of prototroch, metatroch and adoral ciliary zone had become functional in feeding. Furthermore, the theory neither accounts for the complicated shape of the second micromere quartet (figure 2*c,d*), from which the band of compound cilia develops, nor for the origin of the tubular gut with mouth and anus (or for the shape of the ventral CNS).

## 4. Special types of trochophora larvae

The plethora of different larval types in the spiralian phyla shows that this larval type can be modified in many different ways. The rostraria larva of amphinomid annelids (figure 3*f*) has the lateral parts of the feeding apparatus extended onto long tentacles; it swims with a telotroch [19]. Veliger larvae of gastropods and bivalves have the feeding apparatus extended on the edge of a velum, and some entoproct larvae have the lateral sides of the body with the feeding structures expanded almost like a molluscan velum (figure 3*g*). Lecithotrophic larvae lack the metatroch but may have both prototroch and metatroch or only the prototroch. Feeding and non-feeding pericalymma larvae [34] of both annelids and molluscs have the hyposphere differentiating inside a deep invagination (figure 3*h,i*). Direct developing annelids have the spiral cleavage pattern, but may lack the ciliary bands. The spiralian cleavage pattern has been lost in the cephalopods, which have a discoidal cleavage.

## 5. Conclusion

There appears to be complete congruence between the trochaea theory's hypothesized development, shape and function of prototroch, adoral ciliary zone, metatroch, gastrotroch and telotroch, of the tubular gut, and of the ventral CNS (figure 2). It therefore appears highly probable that the trochophora larva evolved through modifications of the trochaea, which divided the blastopore through lateral blastopore lip fusion, i.e. amphistomy, with the circumblastoporal archaeotroch, consisting of compound cilia, becoming differentiated into the prototroch and metatroch around the mouth and the telotroch around the anus. The perioral field of separate cilia of the trochaea differentiated into the adoral ciliary field between prototroch and metatroch and the gastrotroch along the ventral midline. It has been shown that every intermediate stage in this evolutionary scenario could have been viable and that each step could have conferred an adaptational advantage [3].

The alternative theory of Ivanova-Kazas is not supported by any cell-lineage study, and the proposed evolution of the downstream-collecting ciliary system goes through stages, which are definitely non-adaptive. Furthermore, it does not account for the evolution of the convoluted shape of the bands of compound cilia situated at the blastopore edge (a cell-lineage seems impossible to envisage), for the characteristic ventral CNS, or of the tubular gut.

It further appears that the trochophora larva represents the larval type of the spiralian ancestor. It may be ancestral to the whole Protostomia and perhaps even to the Eubilateria [42].

## Data accessibility

This article has no additional data.
